# Characterizing the Causal Pathway From Childhood Adiposity to Right Heart Physiology and Pulmonary Circulation Using Lifecourse Mendelian Randomization

**DOI:** 10.1161/JAHA.123.030453

**Published:** 2024-03-08

**Authors:** Genevieve M. Leyden, Helena Urquijo, Alun D. Hughes, George Davey Smith, Tom G. Richardson

**Affiliations:** ^1^ MRC Integrative Epidemiology Unit (IEU), Population Health Sciences, Bristol Medical School, University of Bristol Bristol UK; ^2^ Bristol Medical School: Translational Health Sciences, Dorothy Hodgkin Building University of Bristol Bristol UK; ^3^ MRC Unit for Lifelong Health and Ageing at UCL, Department of Population Science and Experimental Medicine Institute of Cardiovascular Science, University College London London UK

**Keywords:** cardiac physiology, childhood adiposity, lifecourse epidemiology, Mendelian randomization, vascular structure, Genetic, Association Studies, Epidemiology, Cardiovascular Disease, Obesity

## Abstract

**Background:**

Observational epidemiological studies have reported an association between childhood adiposity and altered cardiac morphology and function in later life. However, whether this is due to a direct consequence of being overweight during childhood has been difficult to establish, particularly as accounting for other measures of body composition throughout the lifecourse can be exceptionally challenging.

**Methods and Results:**

In this study, we used human genetics to investigate this using a causal inference technique known as lifecourse Mendelian randomization. This approach allowed us to evaluate the effect of childhood body size on 11 measures of right heart and pulmonary circulation independent of other anthropometric traits at various stages in the lifecourse. We found strong evidence that childhood body size has a direct effect on an enlarged right heart structure in later life (eg, right ventricular end‐diastolic volume: β=0.24 [95% CI, 0.15–0.33]; *P*=3×10^−7^) independent of adulthood body size. In contrast, childhood body size effects on maximum ascending aorta diameter attenuated upon accounting for body size in adulthood, suggesting that this effect is likely attributed to individuals remaining overweight into later life. Effects of childhood body size on pulmonary artery traits and measures of right atrial function became weaker upon accounting for adulthood fat‐free mass and childhood height, respectively.

**Conclusions:**

Our findings suggest that, although childhood body size has a long‐term influence on an enlarged heart structure in adulthood, associations with the other structural components of the cardiovascular system and their function may be largely attributed to body composition at other stages in the lifecourse.

**Nonstandard Abbreviations and Acronyms**
ALSPAC: Avon Longitudinal Study of Parents and Children
HUNT: Trøndelag Health Study
MR: Mendelian randomization
UKB: UK Biobank

**Clinical Perspective**

**What Is New?**

- This Mendelian randomization study estimated the genetically predicted effect of childhood body size on various measures of right heart structure, function, and pulmonary circulation taken during adulthood.
- Evidence of an independent effect of childhood body size on adulthood right heart structure was found even after accounting for adulthood body size, fat‐free mass, and height.

**What Are the Clinical Implications?**

- These findings suggest that an individual's body size during childhood may have long‐term consequences for right heart structure that persist throughout the lifecourse.
- Conversely, associations between childhood body size and other right heart and pulmonary circulation traits assessed are likely attributed to other traits throughout the lifecourse, such as an individual's body size in adulthood.
- Further research is necessary to determine whether there is a clear pathophysiological connection between childhood body size, right heart structure, and risk of cardiovascular disease in later life.

Childhood obesity has been associated with alterations in cardiac and vascular structure and function in adulthood by conventional epidemiological studies, although establishing whether this is due to a causal relationship is challenging, particularly due to confounding factors throughout the lifecourse.^1^ Furthermore, determining whether adiposity has a direct influence on heart structure and function during the developmental stages of life (ie, which persists into adulthood), as opposed to these associations being attributed to a long‐term consequence of remaining overweight into adulthood, is likewise difficult to disentangle.

These questions motivated our previous research in which we applied a causal inference technique known as Mendelian randomization (MR) to investigate the effect of childhood body size on 4 measures of left ventricular structure and function.^2^ MR leverages genetic variants as instrumental variables to estimate the causal effect of modifiable lifestyle risk factors on complex traits and disease outcomes.^3^, ^4^ As these genetic variants are allocated randomly at birth, estimates derived using MR are typically more robust to confounding and reverse causation compared with those obtained by conventional epidemiological approaches.

Furthermore, by exploiting genetic variants with time‐varying effects on body size, we applied the principles of MR in this previous work to estimate the effect of childhood body size while accounting for the effect of body mass index in adulthood (referred to as “lifecourse MR,”^5^ depicted in Figure 1). Our findings suggested that childhood body size has a direct effect on adulthood measured left ventricular cardiac structure independent of adulthood body size. However, we found limited evidence that body size during either childhood or adulthood influences cardiac function in later life. This warrants further investigation, particularly given the irrefutable evidence from MR and other study designs that find that adiposity increases the risk of cardiovascular disease end points in later life.^6^, ^7^ Moreover, whether this direct effect of childhood body size on cardiac structure is specific to left ventricular traits or whether it is generalizable to other components of the cardiovascular system is unclear.

**Figure 1:**
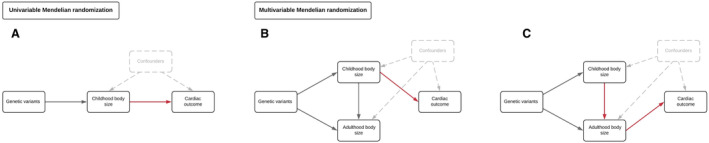
Schematic diagrams depicting different scenarios whereby childhood adiposity may exert an effect on cardiac outcomes in adulthood using a lifecourse Mendelian randomization approach. **A**, Univariable Mendelian randomization was conducted to estimate the “total effect” of childhood body size on cardiac outcomes in adulthood. **B**, Multivariable Mendelian randomization was subsequently applied to estimate “direct effects” of childhood body size on cardiac outcomes (ie, an effect independent of adulthood body size), as well as simultaneously estimating (**C**) “indirect effects,” which are mediated along the causal pathway involving adulthood body size.

In the current study, we applied lifecourse MR to separate the direct and indirect effects of childhood body size on 11 measures relevant to the right heart and pulmonary circulation, encompassing the right atrium, right ventricle, pulmonary artery, and aorta. After initially accounting for adulthood body size in our multivariable model, we next evaluated whether other measures of lifecourse body composition may be responsible for childhood body size effects (ie, childhood height, birth weight, and adulthood fat‐free mass). In doing so, we sought to develop granular insight into the lifelong consequences of childhood body size on cardiovascular system physiology.

## Methods

### Data Availability

All individual‐level data analyzed in this study can be accessed via an approved application to the UK Biobank (UKB) study (https://www.ukbiobank.ac.uk/enable‐your‐research/apply‐for‐access).

#### Data Resources

##### Genetic Instruments for Measures of Lifecourse Body Composition

A flowchart illustrating the stepwise approach conducted in this study can be found in Figure S1. Full details on the instruments derived for this MR study have been reported in detail previously,^2^ as well as annotating the genetic loci for instrumental variables. Briefly, genome‐wide association studies were conducted on 407 741 UKB participants (mean age, 56.5 [range, 40–69] years) who had their current body mass index measured at a clinic visit as well as reporting their body size at age 10. For this childhood measure, UKB participants were asked, “When you were 10 years old, compared with average, would you describe yourself as thinner, plumper, or about average?” To account for possible limitations of using perceived body size rather than a measured variable, genetic variants robustly associated with these measures were identified from these analyses (based on *P*<5×10^−8^), and the resulting genetic score was validated using measured childhood body mass index in ALSPAC (Avon Longitudinal Study of Parents and Children),^5^ the Young Finns Study,^8^ and HUNT (Trøndelag Health Study).^9^ These analyses found that these instruments are capable of separating effects of body size during childhood and adulthood. Furthermore, these instrument sets have recently been validated using detailed dual energy x‐ray absorptiometry–assessed measures of anthropometry, which found that the childhood instruments are much stronger predictors of fat mass compared with lean mass.^10^ Additional details on instrument derivation and validation analyses can be found in Data S1. All data analyzed in this study were summary‐level based and obtained from the UKB study. UKB obtained ethics approval from the Research Ethics Committee (REC; approval number: 11/NW/0382) and informed consent from all participants enrolled in this study.

In this study, we also incorporated genetic instruments for other measures of body composition at different stages in the lifecourse into analyses to evaluate the robustness of our findings for childhood body size. These were childhood height (n=408 516) (ie, to further evaluate that estimates were attributed to adiposity rather than simply being larger in childhood), birth weight (n=230 263) and adulthood fat‐free mass index (n=401 757), which were also derived in the UKB through genome‐wide association studies. UKB individuals who attended the imaging substudy were excluded from these genome‐wide association studies to ensure that the exposure and outcome data sets in our analyses did not include overlapping participants, which has been shown to potentially bias findings in MR studies.^11^ Further details on all instruments derived in this work can be found in Tables S1 to S8.

##### Genetic Estimates of Magnetic Resonance Imaging–Assessed Measures of Cardiac Structure and Function

Genome‐wide effect estimates on 11 measures of cardiovascular structure and function were obtained from previous studies conducted genome‐wide association studies of cardiac magnetic resonance imaging (MRI) traits taken during the UKB imaging substudy.^12^, ^13^ These measures were obtained after 2014, whereas data on exposures in this study were collected between 2006 to 2010 from the UKB. Full details of these traits are described in their corresponding publications, which included excluding participants with diagnoses of heart failure, atrial fibrillation and myocardial infarction. Broadly, these measures can be grouped into measures of right heart structure (ie, right ventricular end diastolic volume, right ventricular end‐systolic volume, and right ventricular stroke volume), right heart function (ie, right ventricular ejection fraction and right atrium fractional area change), the pulmonary artery (ie, short‐axis pulmonary artery, short‐axis pulmonary artery in diastole, short‐axis pulmonary artery strain, and pulmonary root diameter), as well as maximum ascending thoracic aorta diameter and pulmonary artery–to–aorta ratio. An overview of these 11 cardiac outcome traits can be found in Table S2.

#### Statistical Analysis

##### Univariable Mendelian Randomization

We first applied 2‐sample univariable MR to estimate the total effect of genetically predicted childhood body size on each of the cardiac MRI–derived measures in turn (Figure 1A). Conducting MR in a 2‐sample setting permits the use of summary‐level data using estimates from genetic variants derived in 2 separate samples (ie, measures of actual body size in individual‐level data are not required). Estimates are therefore interpreted as genetically predicted effects of childhood body size on cardiac outcomes.

This approach was conducted using the random effects inverse variance weighted method as our primary analysis, which accounts for excess heterogeneity across single nucleotide polymorphism–specific estimates and does not affect the relative weights of individual variant estimates.^14^ This was then followed by applying the weighted median method, which assumes that at least half of the single nucleotide polymorphisms in the analysis are valid instruments.^15^ Additionally, we applied the MR‐Egger method, which assumes that genetic variants do not exert their effects on outcomes via pleiotropic pathways (in line with the Instrument Strength Independent of Direct Effect assumption).^16^ Univariable analyses were also undertaken for all other measures of lifecourse anthropometry for comparison with estimates derived in a multivariable setting.

##### Multivariable MR

Multivariable MR permits the independent effects of multiple exposures (eg, childhood and adult body size) to be simultaneously estimated on an outcome (eg, an MRI‐derived cardiac outcome) using instrumental variables for all exposures in the same model.^17^, ^18^ We followed up univariable analyses in this study by applying multivariable MR in a 2‐sample setting to estimate the direct (Figure 1B) and indirect effects (Figure 1C) of childhood body size on each of the MRI‐derived cardiac measures. This analysis was then repeated but replacing adult body size with each of the other measures of lifecourse anthropometry in turn (ie, birth weight, childhood height, and adult fat‐free mass). In doing so, we sought to investigate whether body composition at these other time points in life may be responsible for the effects of childhood body size on cardiac traits found in a univariable setting.

All MR analyses were undertaken in R version 3.5.1 (R Foundation for Statistical Computing, Vienna, Austria) using the “TwoSampleMR”package.^19^ Forest plots in this paper were generated using the R package “ggplot2.”^20^

## Results

### Investigating the Consequences of Childhood and Adult Body Size on Measures of Right Heart Structure and Function

Univariable MR analyses provided strong evidence that childhood body size has a total effect on all 3 measures of adulthood right ventricular structure (eg, right ventricular end‐diastolic volume: inverse variance weighted β=0.34 SD change per change in body size category [95% CI, 0.27–0.41]; *P*=2×10^−19^) based on all 3 MR methods applied (Table S3). The MR‐Egger intercepts for these analyses provided limited evidence of uncorrelated horizontal pleiotropy after accounting for multiple testing (Table S3). Strong evidence for these effects was also found after accounting for adulthood body size using multivariable MR, suggesting that childhood body size has a direct effect on later life right heart structure independent of body size in adulthood (eg, right ventricular end‐diastolic volume: β=0.24 [95% CI, 0.15–0.33]; *P*=3×10^−7^) (Table S4). Childhood body size estimates also remained robust upon accounting for childhood height in the multivariable framework (Table S5), meaning that these effects are likely attributed to body size rather than simply being a larger individual in childhood. Similar conclusions were drawn after accounting for fat‐free mass index (Table S6) and birth weight (Table S7) in turn.

**Figure 2:**
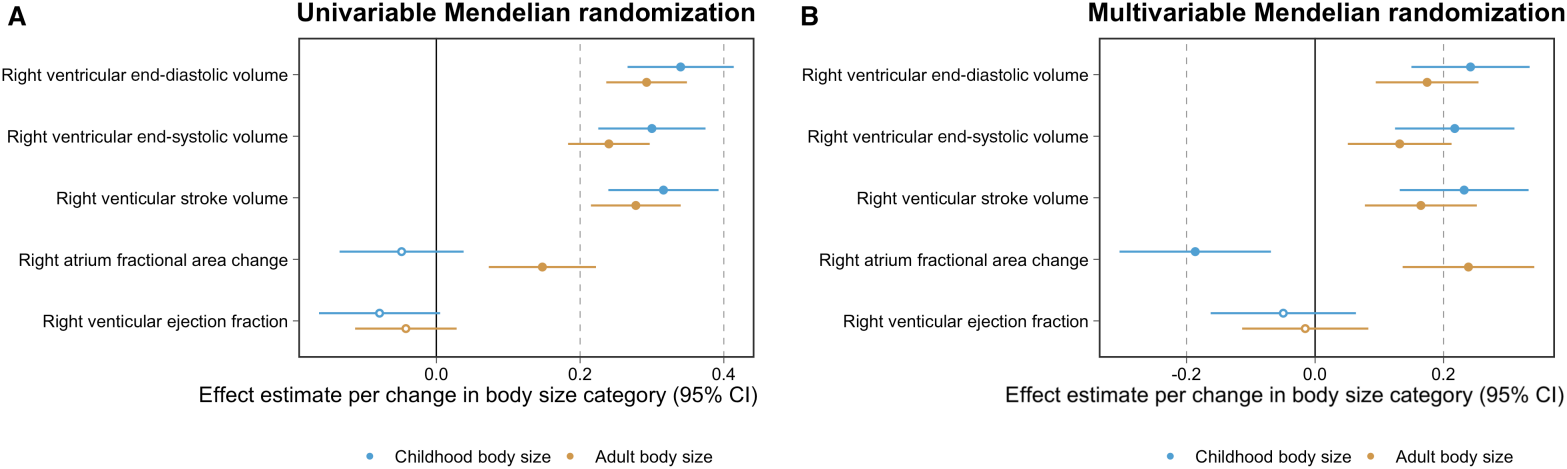
Forest plots illustrating (A) univariable and (B) multivariable Mendelian randomization effect estimates of childhood and adult body size on measures of right heart structure and function. Estimates for childhood (blue) and adult (orange) body size per change in body size category with their corresponding 95% CIs. The data underlying these figures can be found in Table S4.

Conversely, there was weak evidence that body size in childhood influences measures of right heart function in later life (eg, right atrium fractional area change: β=−0.05 [95% CI, 0.14–0.04]; *P*=0.27 [Table S3]), whereas strong evidence was found that adulthood body size increases right atrium fractional area change (β=0.24 [95% CI, 0.14–0.34]; *P*=5×10^−6^). In the multivariable model, the effect estimate for childhood body size provided evidence of an effect on reduced right atrium fractional area change upon accounting for the effect of adult body size (β=−0.19 [95% CI, −0.30 to −0.07]; *P*=2×10^−3^). Additionally, other measures of lifecourse anthropometry provided strong evidence of an effect on right atrium fractional area change in the multivariable model in comparison to childhood body size, such as childhood height (β=−0.14 [95% CI, −0.19 to −0.09]; *P*=1×10^−8^). Findings from all analyses in this section are illustrated in Figure 2.

### Disentangling the Direct and Indirect Effects of Childhood Body Size on Pulmonary Artery Physiology

**Figure 3:**
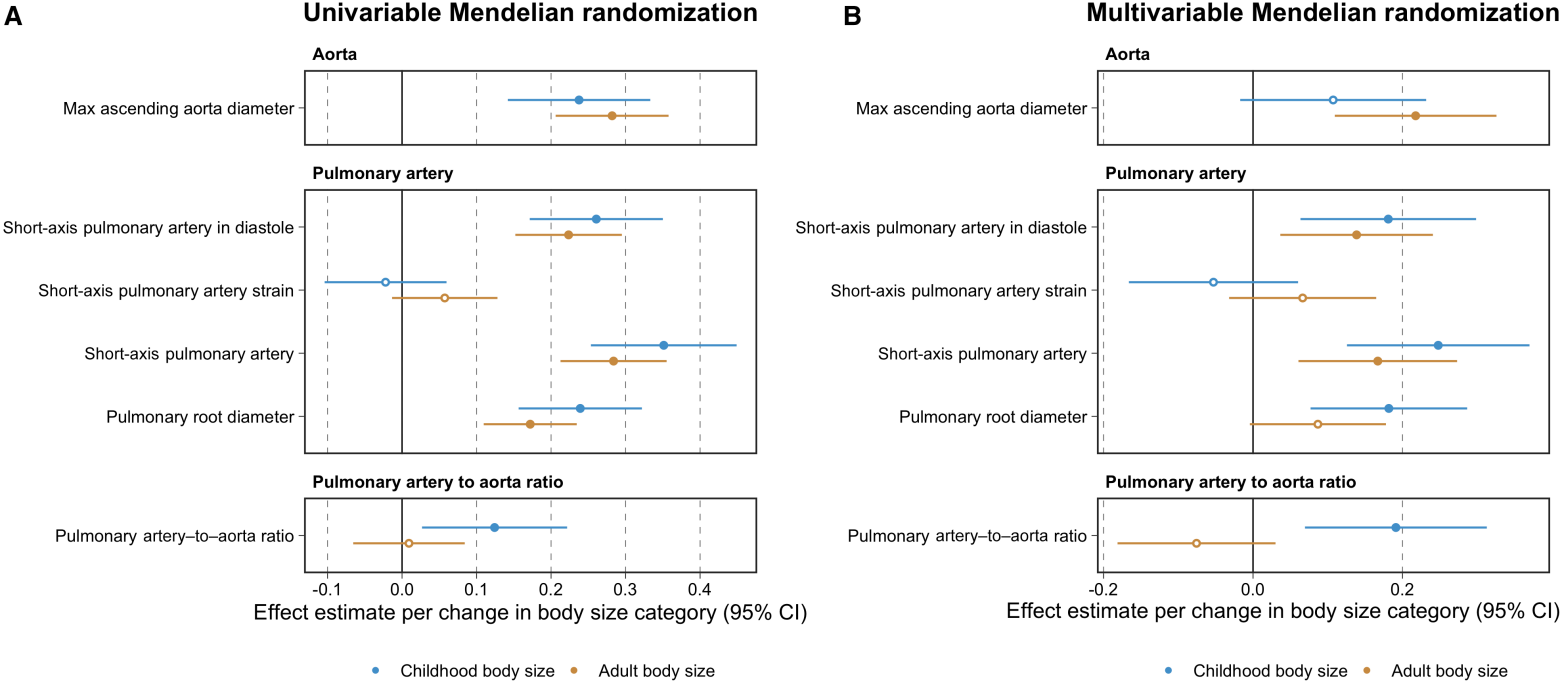
Forest plots illustrating (A) univariable and (B) multivariable Mendelian randomization effect estimates of childhood and adult body size on measures of the pulmonary artery, the thoracic aorta, and their ratio. Estimates for childhood (blue) and adult (orange) body size per change in body size category with their corresponding 95% CIs. The data underlying these figures can be found in Table S4.

Childhood body size provided strong evidence of a total effect on measures of aorta structure (eg, maximum ascending aorta diameter: β=0.24 [95% CI, 0.14–0.33]; *P*=1×10^−6^), pulmonary artery traits (eg, short‐axis pulmonary artery: β=0.35 [95% CI, 0.25–0.45]; *P*=2×10^−12^), and their ratio (β=0.12 [95% CI=0.03–0.22]; *P*=0.01). Effect estimates on aorta structure outcomes attenuated upon accounting for adult body size in the multivariable model (eg, maximum ascending aorta diameter: β=0.11 [95% CI, −0.02 to 0.23]; *P*=0.09), suggesting that being overweight in childhood likely has an indirect effect on later life aorta structure due to a sustained consequence of body size over the lifecourse (Figure 3).

Next, we found that childhood body size typically provided evidence of an independent effect on measures of the pulmonary artery after accounting for adult body size, childhood height, and birthweight in a multivariable setting (eg, short‐axis pulmonary artery after accounting for childhood height: β=0.33 [95% CI, 0.24–0.42]; *P*=2×10^−12^; Figure 3). However, central effect estimates on pulmonary artery traits were reduced by approximately half when accounting for fat‐free mass index in adulthood (eg, short‐axis pulmonary artery: β=0.19 [95% CI, 0.06–0.20]; *P*=0.003), suggesting that lean and muscle mass in adulthood may have a predominating effect on pulmonary artery physiology in later life compared with overall adulthood adiposity.

Finally, while the effect of childhood body size on pulmonary artery–to–aorta ratio remained robust after accounting for adult body size (β=0.19 [95% CI, 0.07–0.31]; *P*=0.002; Figure 3), evidence of an effect became weaker when including other measures of lifecourse anthropometry in the multivariable model (eg, childhood height: β=0.08 [95% CI–0.02 to 0.17]; *P*=0.09). Results for all analyses in this section can be found in Tables S3 through S7. Finally, we found that effect estimates derived in this study on right heart structure traits were broadly comparable with estimates on left heart structure traits (Table S8), reflecting similar pathogeneses between childhood body size and both left and right heart structure traits.

## Discussion

In this study, we applied Mendelian randomization to disentangle the direct and indirect effects of childhood body size on MRI‐derived measures of cardiac physiology. Our results suggest that being overweight in childhood has a direct effect on enlarged right heart structure in later life, independent of other measures of anthropometry at other time points in the lifecourse. Additionally, childhood body size provided evidence of an effect on reduced right atrium fractional area change, increased short‐axis pulmonary traits, and an increased pulmonary artery–to–aorta ratio while accounting for adulthood body size. Extensive analyses of these findings suggested that these effects are likely attributed to an indirect effect via other lifecourse measures of body composition. These inferences would be incredibly challenging to make in an observational epidemiological setting without the aid of human genetics, particularly given that, at a population level, individuals who are overweight in early life typically remain so throughout adolescence and into adulthood.^21^

Our findings indicate that childhood body size may have long‐term and potentially immutable^22^ effects on an enlarged right heart structure in later life, corroborating results from our previous research in which similar conclusions were drawn using left heart structure traits.^2^ A postulated mechanism for this finding is that individuals who are larger in early life typically will have a higher level of adipose tissue and circulating blood volume, both of which have consequences for cardiac structure and function.^23^, ^24^ Taken together with findings from our previous work on left ventricular end‐diastolic and stroke volume,^2^ our results provide evidence that blood volume may be persistently elevated over the lifecourse by childhood body size. These findings are broadly consistent with those identified in an observational setting, which have found an association between obesity and poorer right ventricular structure and elevated pulmonary hypertension, although these studies are unable to reliably separate independent effects of childhood and adult body size, which was appraised in this study using genetic variants.^25^

We also found evidence to suggest that childhood body size may have a long‐term adverse effect on cardiac function independent of adulthood body size on the basis of right atrium fractional area change, which is the percentage change in the right atrium between systole and diastole. That said, findings from the literature suggest that childhood body size has little impact on later life cardiovascular disease upon accounting for the contribution of body size in adulthood.^6^, ^26^ However, these studies mainly focused on outcomes related to systemic circulation, and therefore further investigation into outcomes relevant to cardiac function (such as heart failure) is warranted.

Evidence of a total effect of childhood body size on measures of aorta physiology attenuated upon accounting for body size in adulthood, suggesting that this finding is likely explained by individuals who are larger in childhood typically remaining so throughout the lifecourse. Total effect estimates between childhood body size and pulmonary artery traits most strongly attenuated upon accounting for fat‐free mass in adulthood, which may suggest that lean and muscle mass play a more influential role in pulmonary artery physiology in later life compared with adiposity. Effect estimates between childhood body size and pulmonary artery–to–aorta ratio, which is used as a proxy for pulmonary hypertension risk,^27^ most strongly attenuated when accounting for height in childhood. These findings therefore suggest that being an overall larger individual in early life may be a stronger indicator of this cardiac trait in comparison with childhood adiposity. However, previous applications of these scores have provided evidence of an indirect effect on outcomes, such as atherosclerosis and heart failure,^6^ in contrast to evidence of a direct effect found in our analyses of right heart structure traits. Taken together, these findings do not indicate a clear pathophysiological connection between childhood body size, right heart structure, and risk of heart failure in later life. Further research is therefore necessary to explore this potential causal pathway and determine whether findings may be of clinical relevance.

While our lifecourse MR framework provides a powerful framework to separate direct and indirect effects of childhood body size on disease outcomes and traits, we note that further research is required to pinpoint the critical windows in the lifecourse when changes to cardiac physiology may become irreversible. In addition, our study benefitted from the largest study to date with MRI‐derived measures of cardiac physiology made possible by the UKB study. That said, the subsample of individuals who attended the UKB imaging clinic have been reported to have a “healthy bias,”^28^ which is a caveat that comes from biobank‐level scale data collection. Likewise, the UKB participants were predominantly born in the 1940s, 1950s, and 1960s, meaning that effect estimates derived in this study may not directly translate to individuals born in more recent decades where factors such as the prevalence of childhood obesity are different.

Furthermore, we did not evaluate sex differences between body size and cardiac traits in this study due to data availability, which should therefore be the focus of future investigations. Finally, although our findings are consistent with our previous research regarding left heart structure traits, we were unable to assess whether effects of childhood body size act independently on left and right heart traits. This should therefore also be the aim of future research once larger sample sizes of MRI‐derived cardiac traits facilitate a larger number of genetic loci to analyze within a multivariable MR framework.

In conclusion, findings from this study support those from our previous investigations, suggesting that body size in early life has a lifelong influence on heart structure. Conversely, associations between childhood body size and the other measures of cardiac physiology assessed in this study are likely attributed to measures of body composition over the lifecourse. Future research should focus on the downstream consequences of adiposity on cardiac morphology and how these changes contribute to overall cardiovascular disease risk.

## Sources of Funding

This work was supported by the Integrative Epidemiology Unit, which receives funding from the UK Medical Research Council and the University of Bristol (MC_UU_00011/1 and MC_UU_00032/3). H.U. is supported by a BHF studentship (FS/17/60/33474).

## Disclosures

T.G. Richardson is an employee of GlaxoSmithKline outside of this work. The remaining authors have no disclosures to report.

## Supporting information

Data S1
Figure S1

Tables S1–S8
