# Supplementary material for: Characterizing the Causal Pathway From Childhood Adiposity to Right Heart Physiology and Pulmonary Circulation Using Lifecourse Mendelian Randomization
Source: J Am Heart Assoc. 2024 Mar 8;13(6):e030453. doi: 10.1161/JAHA.123.030453 (PMC11010002; doi:10.1161/JAHA.123.030453)

SUPPLEMENTAL MATERIAL

Data S1. Supplemental Methods

Methods for lifecourse Mendelian randomization instruments

Full details of instrument derivation used in this study has been described in detail previously (5). GWAS of the childhood and adulthood body size variables in the UK Biobank study were conducted by adjusting for age, sex and genotyping chip using the BOLT-LMM software which generates a genetic relationship matrix between samples to account for relatedness and population stratification. Childhood body size was analysed in it's original form as a 3-tier categorical variable whereas the adulthood body size variable was derived by dichotomizing continuously measured body mass index also as a 3-tier categorical variable with the same proportion as the childhood measure. This was performed for interpretation purposes of coefficients calculated from downstream analyses.

Validation analyses of derived genetic scores from these results were undertaken by comparing their genetic correlations with results from GWAS of measured childhood obesity and adult BMI analysed as a continuous variable. Despite the use of recall data in our study to derive the childhood body size instruments, we found that our results were more strongly correlated with measured childhood obesity (r_g=0.85) compared (5) to adult BMI (rg=0.67). In contrast, our adult body size GWAS was very strongly correlated with the adult BMI findings (rg=0.96) in comparison to their correlation with childhood obesity (rg=0.64). We also previously evaluated the prediction of these scores by pooling together all individual variants into a weight genetic risk score and analysing these against measured BMI in three independent cohorts; the Avon Longitudinal Study of Parents and Children (ALSPAC)(5), the Young Finns Study (8) and the Trøndelag Health (HUNT) study (9)). Additionally, in the original study to use these instruments we undertook simulations to investigate potential misclassification which could be attributed to the recall nature of the childhood body size variable. These suggested that bias from misclassification depends on both the type of misclassification and the size and direction of the effects of both exposures on the outcome. This misclassification only masks an effect of the adulthood score when it acts in the same direction as the childhood body size effect on the outcome. Consequently, we do not believe

that this measurement error is responsible for the evidence of a direct effect of the childhood body size score on outcomes analyses. For full details please review the original study (5).

Table S1 - Instrument characteristics for exposures

Found in Excel sheet 1. An overview of the exposure datasets analysed in this study

Table S2: Characteristics for cardiac physiology outcomes

Found in Excel sheet 2. An overview of the outcome datasets analysed in this study

Table S3: Univariable Mendelian randomization analyses for childhood body size on 11 cardiac traits

Found in Excel sheet 3. Results of univariable Mendelian randomization analyses for childhood and adult body size on cardiac traits

Table S4: Multivariable Mendelian randomization analyses for childhood and adulthood body size on 11 cardiac traits

Found in Excel sheet 4. Results of multivariable Mendelian randomization analyses for childhood and adult body size on cardiac traits

Table S5: Multivariable Mendelian randomization analyses for childhood body size and childhood height on 11 cardiac traits

Found in Excel sheet 5. Results of multivariable Mendelian randomization analyses for childhood and adult height on cardiac traits

Table S6: Multivariable Mendelian randomization analyses for childhood body size and adulthood fat-free mass index on 11 cardiac traits

Found in Excel sheet 6. Results of multivariable Mendelian randomization analyses for childhood body size and adult fat-free mass index on cardiac traits

Table S7: Multivariable Mendelian randomization analyses for childhood body size and birthweight on 11 cardiac traits

Found in Excel sheet 7. Results of multivariable Mendelian randomization analyses for childhood body size and birthweight on cardiac traits

Table S8. A comparison of univariable Mendelian randomization effect estimates on left and right heart structure traits (see Excel file)

Figure S1. Flowchart illustrating the stepwise approach conducted in this study.

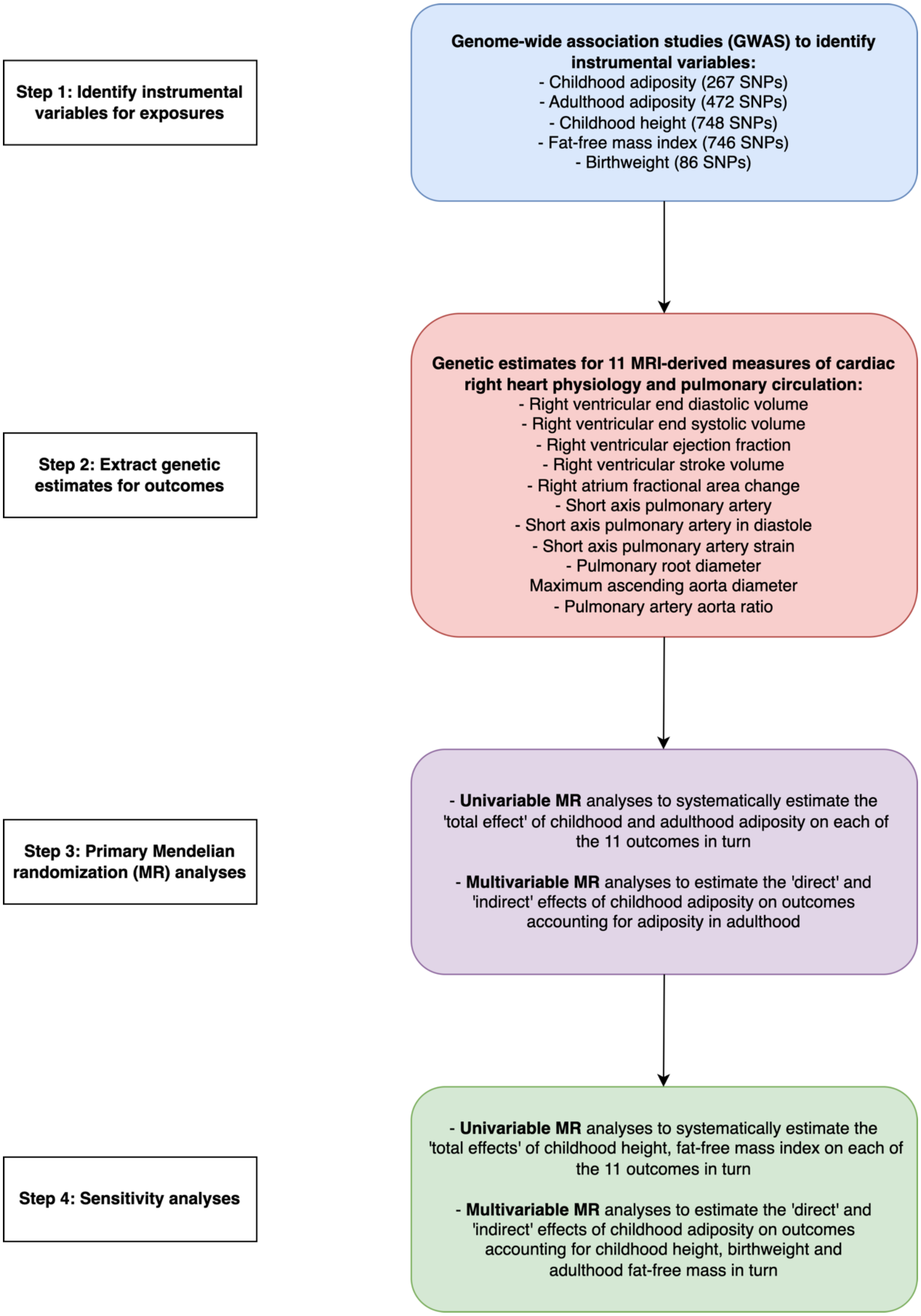

Supplement: Supplementary file 1 — Data S1 Figure S1 [file JAH3-13-e030453-s001.pdf]
